# Trends in utilization of first‐line palliative treatments for anal squamous cell carcinoma

**DOI:** 10.1002/cam4.5126

**Published:** 2022-09-09

**Authors:** Srinidhi J. Radhakrishnan, Suleyman Y. Goksu, Saikripa M. Radhakrishnan, Muhammad S. Beg, Nina N. Sanford, Syed M. Kazmi

**Affiliations:** ^1^ Department of Internal Medicine UT Southwestern Medical Center Dallas Texas USA; ^2^ Department of Internal Medicine, Division of Geriatrics Loyola University Medical Center Hines Illinois USA; ^3^ Division of Hematology and Oncology UT Southwestern Medical Center Dallas Texas USA; ^4^ Science 37 Durham North Carolina USA; ^5^ Department of Radiation Oncology UT Southwestern Medical Center Dallas Texas USA

**Keywords:** anal neoplasms, database, health care utilization, palliative care, quality of life

## Abstract

**Background:**

Anal squamous cell carcinoma patients often present with significant symptoms, including pain, bleeding, and obstructive symptoms. This requires palliation‐directed therapy as a first‐line treatment to alleviate symptoms. The proportion of patients receiving first‐line palliative treatments is unknown. We aimed to study the factors associated with the use of first‐line palliative treatments in stage II–IV anal squamous cell carcinoma patients.

**Methods:**

We used the National Cancer Database to identify adult patients diagnosed with stage II–IV anal squamous cell carcinoma between 2004 and 2016. We performed univariable and multivariable logistic regression analysis to determine the clinical and sociodemographic variables associated with the utilization of palliative treatment in the first‐line setting, including palliative radiotherapy, chemotherapy, surgery, and pain management.

**Results:**

Among 16,944 patients diagnosed with stage II–IV anal squamous cell carcinoma, only a small proportion of 492 (2.9%) required first‐line palliative treatments to control symptoms. The majority of these patients received palliative radiotherapy (32%), followed by palliative surgery (25%), palliative chemotherapy (19%), combination therapies (14%), and pain management (10%). On multivariable analysis, higher stage disease, lower income, Medicare and Medicaid insurance, and life expectancy <6 months were associated with higher odds of use of first‐line palliative therapy.

**Conclusions:**

First‐line use of palliative treatments to control symptoms is needed in a small proportion of anal squamous cell cancer patients. It was utilized in all stages, but it was most frequently observed in patients with stage IV disease and patients with <6 months life expectancy. First‐line palliative therapy was also more frequent in lower‐income patients and patients with Medicare and Medicaid insurance which highlights the disparities in anal cancer management.

## Graphical abstract

Anal squamous cell carcinoma (ASCC) presents with pain, bleeding, and obstructive symptoms, and the proportion of these patients receiving first‐line palliative treatments is unknown. Therefore, patients living in the United States were evaluated using the National Cancer Database. Palliative therapy in ASCC was less utilized and associated with a higher stage of disease and near the end of life.

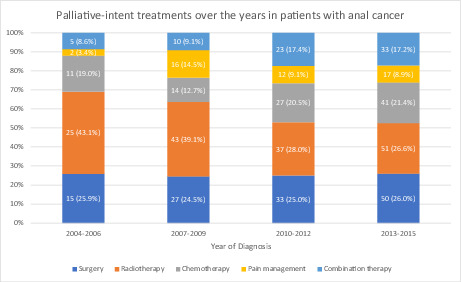

## 1 INTRODUCTION

The incidence of anal squamous cell carcinoma is on the rise worldwide.^1^ The current annual incidence is estimated to be 1/100,000, with women affected more than men, and reports indicating a 2.2% annual increase.^1^, ^2^, ^3^ In patients with localized anal squamous cell carcinoma, the goal of therapy is to achieve a cure while preserving anal function. Treatment is intended to achieve remission at 8–24 weeks with surveillance up to 5 years after completion of therapy. Approximately 10%–20% of patients suffer distant relapse with sites of distant metastases most often affecting para‐aortic nodes, liver, lungs, and skin.^2^ Although localized anal cancer has a 5‐year survival of over 80%, anal cancer with distant metastases has a 5‐year survival rate of less than 35%.^4^

A significant proportion of anal squamous cell cancer patients present with uncontrollable pain and bleeding, requiring initial palliative therapy as a first‐line effort to alleviate symptoms. These therapies or strategies to relieve symptoms may include surgery, radiation therapy, systemic therapy (chemotherapy, hormone therapy, or other systemic drugs), and/or other pain management therapy. These palliative therapies are not used to diagnose or stage the primary tumor, but their main aim is to improve patients’ symptom burden and quality of life.^5^, ^6^, ^7^ The utilization rate of first‐line palliative therapies for anal cancer is unknown. Therefore, the primary objective of this study is to determine the utilization, trends, and factors associated with first‐line palliative therapies in stage II‐IV anal squamous cell carcinoma.

## 2 MATERIALS AND METHODS

The National Cancer Database is one of the largest clinical oncology datasets in the United States, including more than 70% of new cancer cases with demographics, socioeconomic status, tumor characteristics, and treatments. This database provides de‐identified data collected from over 1500 Commission on Cancer (CoC)‐accredited hospitals.^8^

### 2.1 Study population

**FIGURE 1:**
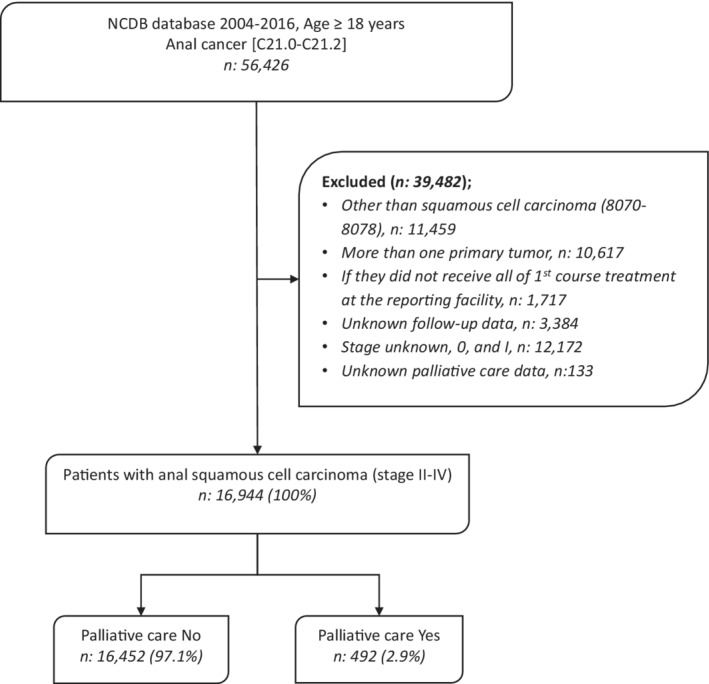
Patient selection diagram for patients with anal squamous cell carcinoma who received palliative care treatment.

We evaluated 16,944 patients diagnosed with anal squamous cell carcinoma between 2004 and 2016 using the ICD‐O‐3/WHO 2008 site recode “C21.0‐C21.2” and ICD‐O‐3 histologic codes “8070–8078”.^9^, ^10^, ^11^, ^12^ Patients equal to and older than 18 years with stage II–IV disease and squamous cell carcinoma histology were included. We chose stage II–IV patients as any such patients can present with severe symptoms that may need first‐line palliative treatments. We excluded patients where follow‐up data and utilization of palliative treatment were unknown. We also excluded patients with secondary tumors and those who did not receive the first line of treatment at the reporting center (N: 39,482) (Figure 1).

### 2.2 Definitions

NCDB defines palliative therapies as surgical procedures, radiation, or systemic therapies provided to prolong the patient's life by controlling symptoms, alleviating pain, or making the patient comfortable. These therapies could be coded as palliative care if used in a first‐line setting and if the procedures removed or modified either primary or metastatic malignant tissue without the goal of staging, diagnosing, or treating the patient. NCDB distinguishes a treatment modality for curative treatment from the same modality used strictly for palliation. Type of palliation directed treatments was defined in NCDB as surgery (including bypass procedures) to alleviate symptoms, radiation therapy, chemotherapy, or other systemic drugs without the intent to treat the primary tumor, pain management therapy, or any combination of surgery, radiation therapy, and/or chemotherapy with or without pain management.^13^ We categorized survival time as <6, 6–24, and >24 months.^13^ The year of diagnosis was stratified as 2004–2006, 2007–2009, 2010–2012, and 2013–2015. We used the 6th and 7th AJCC clinical stage groups to define staging due to no changes between the two editions.^14^, ^15^

### 2.3 Statistical analysis

We used the chi‐square test to compare categorical variables by palliative therapy status. Using the multivariable logistic regression method, we assessed the relationship between palliative therapies and other variables, including survival time. Other variables were age (<49, 50–64, ≥65 years), year of diagnosis, race and ethnicity (non‐Hispanic white, non‐Hispanic black and Hispanic), gender (male, female), facility type (academic/research, non‐academic), facility location, rurality (metropolitan, non‐metropolitan), median income quartiles (<$40,227, $40,227–50,353, $50,354–63,332, ≥$63,333), education level (percentages of patients with no high school level ≥17.6%, 10.9%–17.5%, 6.3%–10.8%, <6.3%), insurance status (uninsured, private insurance, Medicaid, Medicare, other government insurance), travel distance to treatment facility (<12.5, 12.5–49.9, ≥50 miles), Charlson‐Deyo Score (0–2+), and American Joint Committee on Cancer (AJCC) staging. Missing data were handled as an unknown variable and included in multivariable analysis. SPSS version 25.0 was performed for all analyses. *p*‐values <0.05 were considered statistically significant. Due to the use of de‐identified data, the Institutional Review Board at the University of Texas Southwestern deemed this study exempt.

## 3 RESULTS

### 3.1 Study cohort: baseline characteristics

The NCDB database incorporates patients over the age of 18 from 2004 to 2016. Among 16,944 patients diagnosed with stage II–IV anal squamous cell cancer, 492 (2.9%) received palliative therapies as the first‐line therapy. Palliative treatments in first‐line were more likely to be used in older age‐group versus younger‐age group (3.8% vs. 2.7%, *p* < 0.001), male gender versus female gender (3.3% vs. 2.7%, *p* = 0.025), and lower‐income level <$40,227 compared to income greater than $63,333 (3.6% vs. 2.2%, *p* < 0.001). In addition, patients who received first‐line palliative therapy had a higher Medicare insurance rate than private insurance (4.0% vs. 1.8%, *p* < 0.001). First‐line use of palliative therapy was higher in stage 4 disease patients as compared to stage 3 and 2 disease groups (17.2% vs. 2.7% vs. 1%, *p* < 0.001, respectively). Similarly, grade 3 and 4 tumors were more frequently in the first‐line palliative therapy group (3.6%, *p* < 0.001), as outlined in Table 1. Also, first‐line palliative therapy use was higher in East North Central USA than in other facility locations (3.4%, *p* = 0.003). There was no significant racial disparity between those who were and were not assigned to first‐line palliative treatments (Table 1). The majority of these patients received palliative radiotherapy (32%), followed by palliative surgery (25%), palliative chemotherapy (19%), combination therapies (14%), and pain management (10%). For stage II and stage III anal squamous cell cancer patients, first‐line palliative surgery (32.1% and 41.8%, respectively) was the most frequently used palliation modality, followed by palliative radiation (29.8% and 29.1%, respectively). For stage IV patients, first‐line palliative treatment was radiation (34.7%), followed by chemotherapy (32.4%), and then surgery (8.7%). These differences were statistically significant (*p* < 0.001).

**TABLE 1:** Baseline characteristics of patients with anal squamous cell carcinoma

Characteristics	Palliative treatment		*p*‐value
	Yes (%) 492 (2.9)	No (%) 16452 (97.1)	
Age at diagnosis			<0.001
≤49	98 (2.7)	3581 (97.3)	
50–64	201 (2.5)	7936 (97.5)	
≥65	193 (3.8)	4935 (96.2)	
Gender			0.025
Male	197 (3.3)	5779 (96.7)	
Female	295 (2.7)	10673 (97.3)	
Race/Ethnicity			0.131
Non‐Hispanic White	370 (2.8)	12735 (97.2)	
Non‐Hispanic Black	70 (3.7)	1804 (96.3)	
Hispanic	20 (2.4)	808 (97.6)	
Other/unknown	32 (2.8)	1105 (97.2)	
Year of diagnosis			0.05
2004–2006	58 (2.2)	2608 (97.8)	
2007–2009	110 (2.9)	3649 (97.1)	
2010–2012	132 (2.8)	4507 (97.2)	
2013–2015	192 (3.3)	5688 (96.7)	
Comorbidity score			<0.001
0	358 (2.6)	13211 (97.4)	
1	77 (3.7)	2010 (96.3)	
2+	57 (4.4)	1231 (95.6)	
Facility type			0.231
Community cancer program	44 (2.7)	1572 (97.3)	
Comprehensive community cancer program	182 (2.6)	6693 (97.4)	
Academic/research program	181 (3.2)	5469 (96.8)	
Integrated network cancer program	55 (2.6)	2088 (97.4)	
Facility location			0.003
New England	41 (4.2)	924 (95.8)	
Middle Atlantic	77 (3.1)	2395 (96.9)	
South Atlantic	105 (2.8)	3630 (97.2)	
East North Central	97 (3.4)	2725 (96.6)	
East South Central	32 (2.7)	1154 (97.3)	
West North Central	30 (2.6)	1133 (97.4)	
West South Central	20 (1.8)	1106 (98.2)	
Mountain	21 (2.9)	695 (97.1)	
Pacific	39 (1.9)	2060 (98.1)	
Insurance status			<0.001
Uninsured	39 (3.4)	7390 (96.6)	
Private	138 (1.8)	1117 (98.2)	
Medicaid	68 (3.6)	1820 (96.4)	
Medicare	232 (4.0)	5536 (96.0)	
Medicaid expansion			<0.001
Non‐expansion states	167 (2.7)	6008 (97.3)	
Jan 2014 expansion states	155 (3.0)	4974 (97.0)	
Early expansion states (2010–2013)	50 (1.8)	2797 (98.2)	
Late expansion states (after Jan 2014)	90 (4.2)	2043 (95.8)	
Suppressed for ages 0–39	30 (4.5)	630 (95.5)	
Income			0.001
<$40,227	128 (3.6)	3441 (96.4)	
$40,227–50,353	123 (3.1)	3832 (96.9)	
$50,354–63,332	114 (3.0)	3642 (97.0)	
≥$63,333	117 (2.2)	5258 (97.8)	
Education			0.313
≥17.6%	120 (3.3)	3567 (96.7)	
10.9–17.5%	139 (3.0)	4544 (97.0)	
6.3–10.8%	132 (2.8)	4502 (97.2)	
<6.3%	93 (2.5)	3584 (97.5)	
Rurality			0.104
Metropolitan	397 (2.8)	13682 (97.2)	
Non‐metropolitan	85 (3.4)	2405 (96.6)	
Travel distance			0.017
<12.5 miles	292 (2.7)	10332 (97.3)	
12.5–49.9 miles	144 (2.9)	4848 (97.1)	
≥50 miles	53 (4.2)	1219 (95.8)	
Stage			<0.001
II	84 (1.0)	8494 (99.0)	
III	189 (2.7)	6907 (97.3)	
IV	219 (17.2)	1051 (82.8)	
Grade			<0.001
I	26 (1.7)	1537 (98.3)	
II	165 (2.6)	6239 (97.4)	
III–IV	165 (3.6)	4429 (96.4)	
Treatment types			
Chemoradiation	—	12876 (95.0)	
Radiation only	—	671 (5.0)	
Palliative treatment modality			
Surgery	125 (25.4)	—	
Radiation	156 (31.7)	—	
Systemic	93 (18.9)	—	
Pain management	47 (9.6)	—	
Combination	71 (14.4)	—	
Survival (months)			<0.001
<6	127 (8.2)	1415 (91.8)	
6–24	228 (4.9)	4437 (95.1)	
>–24	137 (1.3)	10600 (98.7)	

### 3.2 Trends of use of first‐line palliative therapies

**FIGURE 2:**
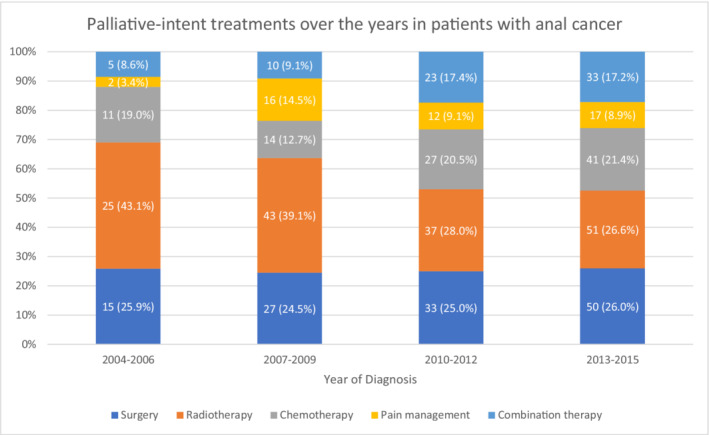
First‐line Palliative treatments in anal squamous cell cancer patients stratified by type of therapy.

The use of first‐line palliative treatments in anal squamous cell cancer increased from 2.2% in 2004–2006 to 3.3% in 2013–2015 (*p* = 0.005). Within the subset of the population receiving first‐line palliative treatments, the surgery rate increased over the years, whereas radiotherapy utilization decreased (Figure 2). Most patients who utilized first‐line palliative therapies had Medicare insurance; however, this trend decreased over time (*p* < 0.001). On the contrary, the utilization trend increased among patients with Medicaid insurance. First‐line palliative treatment was utilized more frequently in male patients, and this difference increased over recent years (*p* = 0.025). States with early adoption of Medicaid expansion had statistically significantly lower use of first‐line palliative therapies compared to late expansion states (*p* < 0.001). The use of first‐line palliative therapy was not different based on treatment in community cancer programs compared to comprehensive cancer programs or academic medical centers (*p* = 0.234). It was more frequently used within 6 months of patient death, with 8.2% of patients with survival <6 months versus 1.3% in patients with survival >24 months (*p* < 0.001). No significant difference was noted regarding race and utilization of first‐line palliative treatments over time.

### 3.3 Logistic regression

Multivariable logistic regression analysis was performed to assess the relationship between palliative therapies and baseline variables after controlling for differences between the cohorts. On multivariable analysis, patients with stage 4 disease (vs. stage 2 disease) (OR 15.8 [12.0–20.7], *p* < 0.001) and Medicare insurance (vs. private insurance) (OR 1.89 [1.43–2.50], *p* < 0.001) were the variables that maintained significance, and they were more likely to receive palliative therapy. Patients with the highest income strata (greater than $63,333) were less likely to receive first‐line palliative therapies as compared to patients with the lowest income strata (<$40,227) (OR 0.72 [0.54–0.95], *p* = 0.022). In addition, patients with a life expectancy ≥24 months were less likely to receive palliative therapy than patients with a life expectancy <6 months (OR 0.29 [0.22–0.38], *p* < 0.001) (Table 2).

**TABLE 2:** Multivariable logistic regression analysis to assess the relationship between first‐line palliative therapy and other variables

Characteristics	OR (95% CI)	*p‐*value
Age		
<50	Ref	
50–64	1.06 (0.79–1.42)	0.69
≥65	1.25 (0.89–1.77)	0.20
Gender		
Male	Ref	
Female	0.95 (0.78–1.16)	0.62
Race/Ethnicity		
Non‐Hispanic White	Ref	
Non‐Hispanic Black	1.07 (0.80–1.44)	0.63
Hispanics	0.65 (0.40–1.05)	0.08
Other/unknown	1.01 (0.68–1.49)	0.96
Comorbidity score		
0	Ref	
1	1.39 (1.07–1.82)	0.015
2+	1.29 (0.94–1.77)	0.11
Facility type		
Academic/research	Ref	
Non‐academic	0.85 (0.69–1.05)	0.14
Unknown	1.75 (1.08–2.82)	0.022
Travel distance		
<12.5 miles	Ref	
12.5–49.9 miles	1.13 (0.90–1.41)	0.31
≥50 miles	1.36 (0.95–1.96)	0.09
Unknown	1.32 (0.32–5.45)	0.71
Income		
<$40,227	Ref	
$40,227–50,353	0.96 (0.73–1.26)	0.76
$50,354–63,332	0.96 (0.72–1.27)	0.75
≥$63,333	0.72 (0.54–0.95)	0.022
Unknown	0.99 (0.46–2.13)	0.98
Insurance status		
Private	Ref	
Uninsured	1.33 (0.91–1.96)	0.14
Medicaid	1.46 (1.07–2.01)	0.018
Medicare	1.89 (1.43–2.50)	<0.001
Other Government/unknown	1.19 (0.67–2.11)	0.55
Rurality		
Metropolitan	Ref	
Non‐metropolitan	0.99 (0.74–1.34)	0.98
Unknown	1.03 (0.51–2.08)	0.93
Stage		
II	Ref	
III	2.55 (1.96–3.31)	<0.001
IV	15.8 (12.0–20.7)	<0.001
Grade		
I	Ref	
II	1.18 (0.77–1.82)	0.45
III–IV	1.40 (0.90–2.16)	0.13
Unknown	1.33 (0.86–2.07)	0.20
Survival (months)		
<6	Ref	
6–24	0.74 (0.58–0.95)	0.016
≥24	0.29 (0.22–0.38)	<0.001

Abbreviations: CI, confidence interval; OR, odds ratio.

## 4 DISCUSSION

Among patients with Stage II–IV anal squamous cell cancer, a small proportion of patients (2.9%) received upfront palliative therapies as the first course of treatment. This remains an understudied population, and ours is the first report of first‐line palliative treatment use in anal squamous cell cancer.

Anal squamous cell carcinoma usually presents with a significant symptom burden at the time of presentation, including intractable pain (30–50%), discharge (50%), bleeding (45%), obstipation, and obstructive symptoms (30%); it is estimated that only 20% of cases may be asymptomatic.^16^ Anal cancer patients who receive definitive intent chemoradiation therapy to treat primary tumors still suffer from long‐term poor health‐related quality of life compared to an age‐matched healthy control group.^17^ Long‐term side effects include sexual dysfunction, venous thrombosis, proctitis, tenesmus, anal stenosis, and bladder dysfunction. The results indicate that a small percentage of anal squamous cell carcinoma patients present with extreme symptoms requiring the first‐line therapy to focus on immediate palliation‐directed therapies before any definitive therapy.

As expected, the palliative therapies as first‐line treatment were most frequent in the later‐stage patients, older age group, and patients with high co‐morbidities. The life expectancy of fewer than 6 months was clearly associated with the utilization of palliative therapy. However, this analysis highlighted several social determinants of health associated with receiving first‐line palliative therapies. Individuals from lower‐income strata and with Medicare and Medicaid insurance had higher utilization rates of palliative treatments in front‐line settings. Our previous work in early‐stage anal cancer also showed that socioeconomic disparities adversely affect treatment and outcomes in anal cancer.^18^ Race did not have a significant role in the current cohort. Consistently, Medicare and Medicaid patients had higher use of first‐line palliative therapies, which was also noted in the other studies conducted in similar populations in colorectal and pancreatic cancer patients.^5^, ^13^

The current results showed that radiation therapy was the most frequently used first‐line palliative treatment, with an estimated one‐third of the cohort receiving it, followed by surgery which was used in a quarter of patients. Radiation therapy has a central role in managing anal squamous cell cancer and effectively controls local symptoms such as uncontrolled bleeding and uncontrolled pain. First‐line palliative radiation use was more frequent in stage IV as compared to stage II and III. This indicates the clinical need to palliate symptoms in an overly symptomatic patient and allow early initiation of systemic therapy. The first‐line palliative surgical approaches in anal squamous cell cancer generally include the creation of a colostomy and excision of the anal mass with the aim to improve constipation or obstipation symptoms and treat impending or overt intestinal obstruction. In this cohort, first‐line palliative surgery was more frequently used in stage II and III patients, which indicates that they presented with significant obstructive symptoms and could not undergo the standard curative approach of combined chemoradiation without first surgical diversion. Clinically, stage II and III anal squamous cell cancer patients are treated with concurrent chemoradiation therapy with curative intent. However, it can take many weeks to work, so first‐line palliative surgery likely allowed a rapid relief of obstructive symptoms, subsequently allowing concurrent chemoradiation to treat the localized disease. Even though survival outcomes were not the main focus, we looked at the median overall survival of our cohort and with each modality (results not reported) and, as anticipated, we noticed that our cohort had shorter survival of 14 months as compared to historically reported data in anal squamous cell cancer, but better with surgery as compared to radiation, likely reflecting this pattern of first‐line palliative treatment utilization based on the stage of cancer.

We acknowledge several limitations, particularly with this study being retrospective, which carries the risk of selection bias. NCDB may have different coverage exist, especially for certain cancers and geographic regions but these gaps for case coverage narrow with increased facility accreditation.^19^, ^20^ Even though this study uses a large database, the population utilizing first‐line palliative therapy was low. The use of large databases can cause potential coding errors. This study only incorporates those receiving palliative treatment as the first‐line therapy, so patients requiring palliative therapies later in their treatment course remain unknown. NCDB also does not have information regarding certain variables that could affect overall survival, including the type of chemotherapy regimens and response rates. Furthermore, information regarding the complication rates of palliative‐intent therapies and HIV/HPV was also not captured in the NCDB database. These factors can potentially affect the survival and quality of life outcomes but could not be incorporated into the study due to the limitations of the database.

## 5 CONCLUSIONS

Between the years 2004–2016, the use of first‐line palliative therapy in anal squamous cell cancer patients was observed in a small proportion of patients. The first‐line palliative modalities were used more frequently in males, older adults, and patients with high co‐morbidities. Its use was also more common in patients with lower‐income strata and those with Medicare and Medicaid insurance. This study highlights and fills the gap of clinically relevant but understudied questions about the need for first‐line palliative therapies.

## AUTHOR CONTRIBUTIONS

Conception, design, analysis, and interpretation of data: Radhakrishnan, Goksu, Radhakrishnan, Sanford, Beg, And Kazmi. Drafting, revision, and final approval of manuscript: All authors.

## CONFLICT OF INTEREST

None.

## ETHICS STATEMENT

Due to the use of de‐identified data, the Institutional Review Board at the University of Texas Southwestern deemed this study exempt.

## Data Availability

The National Cancer Data Base (NCDB) is a joint project of the Commission on Cancer of the American College of Surgeons and the American Cancer Society. This study used de‐identified NCDB data. They are not responsible for the statistical validity of the data analysis or the conclusions derived by the authors. Some restrictions apply to these data's availability; therefore, the data is not publicly available.
